# Cx43 Expression Correlates with Breast Cancer Metastasis in MDA-MB-231 Cells In Vitro, In a Mouse Xenograft Model and in Human Breast Cancer Tissues

**DOI:** 10.3390/cancers11040460

**Published:** 2019-04-01

**Authors:** Jalal M. Kazan, Jamal El-Saghir, Jessica Saliba, Abdullah Shaito, Nour Jalaleddine, Layal El-Hajjar, Sara Al-Ghadban, Lamis Yehia, Kazem Zibara, Marwan El-Sabban

**Affiliations:** 1 Department of Anatomy, Cell Biology and Physiological Sciences, Faculty of Medicine, American University of Beirut, Beirut 1107 2020, Lebanon; jalal.kazan@mail.mcgill.ca (J.M.K.); jae13@mail.aub.edu (J.E.-S.); sara.ghadban@gmail.com (S.A.-G.); lamis.yahya@case.edu (L.Y.); 2 Department of Biology, Faculty of Sciences, Lebanese University, Hadath 1003, Lebanon; jessicasaliba@hotmail.com; 3 Department of Biological and Chemical Sciences, Faculty of Arts and Sciences, Lebanese International University, Beirut 1105, Lebanon; abdshaito@gmail.com; 4 Department of Biological and Environmental Sciences, Faculty of Science, Beirut Arab University, Beirut 1107 2809, Lebanon; nourjalaleddine@hotmail.com (N.J.); layal--hajjar@hotmail.com (L.E.-H.); 5 PRASE and Biology Department, Faculty of Sciences, Lebanese University, Hadath 1003, Lebanon

**Keywords:** breast cancer, connexin43, metastasis, triple negative breast cancer, epithelial-to-mesenchymal transition, EMT

## Abstract

Connexins regulate multiple cellular functions and are considered tumor suppressors. Connexin43 (Cx43) is frequently down-regulated in breast tumors. However, Cx43 regulation during cancer onset and metastasis is complex and context-dependent. We investigated the effect of Cx43 over-expression or knock-down on the metastatic potential of MDA-MB-231 breast cancer cells in vitro and in vivo and in human breast cancer tissues. MDA-MB-231 cells over-expressing (Cx43D) or down-regulating Cx43 (shCx43) were generated and used in proliferation, migration, and invasion assays. The regulation of genes/proteins implicated in progression, invasion and metastasis was assessed in vitro and in immune-compromized mice injected with MDA-MB-231, Cx43D or shCx43 cells. Primary tumor onset/growth, metastasis and overall survival of these animals was monitored and evaluated. In addition, Cx43 expression in human breast carcinoma samples was assessed by qPCR. Cx43 over-expression increased protein levels of epithelial markers E-cadherin and zonula occludens 1 expression and resulted in the sequestration of β-catenin at the cell membrane, while Cx43 knock-down induced protein expression of the mesenchymal marker N-cadherin and an increased invasive potential of shCx43 cells. In vivo, in mice xenografted with breast cancer cells, Cx43 over-expression decreased tumor volume, attenuated cell metastasis to lungs and liver and increased overall mice survival. Importantly, the expression of Cx43 in triple negative human breast cancer tissues is also down-regulated. Collectively, Cx43 over-expression induced an epithelial-like phenotype in MDA-MB-231 cells and suppressed tumor growth and metastasis to secondary organs in vivo. In contrast, Cx43 knock-down in MDA-MB-231 cells induced a mesenchymal phenotype with increased cell invasion leading to an enhanced metastatic phenotype. These data provide evidence for a pivotal role of Cx43 in breast cancer metastasis and support the potential targeting of connexins in breast cancer therapy.

## 1. Introduction

Metastasis, the colonization of tumor cells into selective secondary organ sites from a primary location [1,2,3], accounts for more than 90% of cancer-related mortality. It is a multistep process by which cancer cells detach from the primary tumor, invade the surrounding microenvironment, intravasate, survive the hemodynamics of circulation and extravasate into secondary organs [1,2]. Cancer metastasis depends on cell-cell and cell-matrix interactions, angiogenesis, epithelial to mesenchymal transition (EMT) and soluble factors, such as vascular endothelial growth factor (VEGF) [1,2,4,5,6]. During cancer progression, hypoxia-inducible factors (HIFs) are induced leading to the subsequent expression of angiogenic factors, including VEGF. Cancer cells communicate with endothelial cells of target organs through both paracrine stimulation and gap junctions (GJs), causing a breach in the endothelial barrier, hence allowing cancer cell extravasation [6,7]. GJs are clusters of channels made up of connexin proteins; they directly link the cytoplasm of two adjacent cells and play an essential role in tissue homeostasis, cell proliferation and differentiation [8]. Regulation of connexins expression and their assembly into GJs is complex and is spatially and temporally regulated during different stages of tumorigenesis in a context-dependent manner [9,10]. Previous studies have demonstrated the involvement of connexin 43 (Cx43) in breast cancer progression [11,12]. However, reports on the modulation of Cx43 levels in early cancer onset and in cancer progression were not conclusive [10,13]. Whereas Connexin43 (Cx43) is down-regulated in many primary tumors [14,15], it is up-regulated during later stages of breast carcinogenesis [16,17,18]. Studies have reported that Cx26 and Cx43 were up-regulated in lymph node metastases [19] and that gap junctional communication is essential in breast cancer progression at extravasation sites [18]. In addition, over-expression of connexins reduced the proliferation and malignant phenotype of breast cancer cells in vitro and in vivo [11,20,21,22,23]. A recent study reported that functional Cx43 channel-rich biovesicles reduced cell migration of recipient breast cancer cells [24]. Accordingly, connexin down-regulation caused an aggressive phenotype in breast cancer cells in vitro, where cell proliferation, migration and angiogenesis-related genes were induced [25]. In vivo, Cx43 mutant mice exhibited mammary gland hyperplasia and lung metastasis [26] whereas Cx26 knockout mice predisposed the mammary gland to chemically-induced tumor formation [27]. In human patients, Cx43 expression was reported to be absent in different types of breast carcinoma [28]. Notwithstanding, other studies have attributed a cancer-promoting role for connexins [19,29,30,31,32,33,34,35,36].

In particular, the role of connexins in the progression of triple negative breast cancer (TNBC), the most aggressive subgroup of breast cancer, is still divisive. This subtype lacks estrogen receptor (ER), progesterone receptor (PR) and human epidermal growth factor receptor 2 (HER2) expression, which renders the patients refractive to endocrine therapy [37]. One study showed that connexins were differentially expressed in breast cancer patients depending on cancer grade and subtype and that Cx43 levels inversely correlated with overall survival of TNBC patients [38].

In this study, we adopted a multifaceted approach to evaluate the role of Cx43 in the progression and metastasis of breast cancer. We have employed an established and well-accepted in vitro cell model of TNBC (MDA-MB-231 cells), an in vivo xenograft model of the same cells as well as archived human breast cancer specimens to assess the implications of Cx43 in metastasis.

MDA-MB-231 cells were modulated to either down-regulate or over-express Cx43 in order to explore the effect of Cx43 protein levels on the invasive and metastatic behavior of these cells in vitro and in a murine xenograft model of TNBC in vivo. The differential expression levels of Cx43 were also assessed in different subtypes of human breast carcinoma specimens, including TNBC, to support the notion that Cx43 may serve as a potential target in novel therapeutic modalities against aggressive breast cancer.

## 2. Results

Three cell lines were used in this study: the parental MDA-MB-231 cells and MDA-MB-231 cells modified to overexpress or down-regulate Cx43.

### 2.1. Validation of Cx43 Knock-Down or Over-Expression in MDA-MB-231 Cells

**Figure 1:**
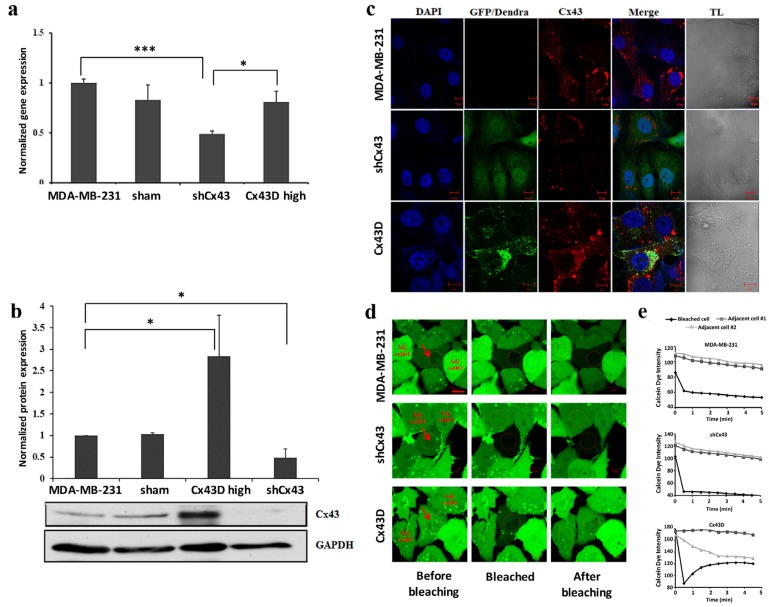
Down-regulation or over-expression of Cx43 in MDA-MB-231 cells. (**a**) Bar graph representing Cx43 mRNA expression in MDA-MB-231, sham cells, shCx43 and Cx43D cells as detected by qPCR and normalized to GAPDH. Results are representative of three independent experiments. (**b**) Western blot of Cx43 protein expression in MDA-MB-231, sham cells, shCx43 and Cx43D cells with densitometry analysis of two independent experiments, after normalization to GAPDH. (**c**) Representative immunofluorescence images of Cx43 expression in parental MDA-MB-231, shCx43 and Cx43D cells. DAPI was used as a nuclear stain and transmitted light (TL) microscopy was used to show cell morphology. GFP/Dendra panel represents the green fluorescence of MDA-MB-231 cells transfected/transduced with shCx43/Cx43D vectors, respectively. Scale bar = 10 µm. (**d**) Representative fluorescence images of FRAP. Red arrows indicate the photobleached cells; ‘Adj. cell#1 and’ ‘Adj. cell#2’ refer to non-photobleached adjacent cells. Scale bar represents 10 µm. (**e**) Quantification of fluorescence intensity of regions of interest (ROIs) relative to adjacent unbleached cells. Values represent the fluorescence intensity (averages ± SD) of each ROI based on several measurements calculated by the Zeiss Zen 2011 software. A minimum of ten different ROIs per condition were analyzed. Sham cells are the GFP-negative cells obtained after sorting of shCx43 or Cx43D cells. * *p* < 0.05; *** *p* < 0.001.

Cx43 expression was assessed in the sorted shCx43 or Cx43D cells. Cx43 expression decreased significantly in shCx43 cells at the transcriptional (*p* < 0.001, Figure 1a) and translational (*p* < 0.05, Figure 1b) levels, as assessed by qPCR, western blotting and by immunofluorescence (Figure 1c). 

No significant change was observed in endogenous Cx43 mRNA levels in Cx43D cells (Figure 1a), using qPCR primers that only detect endogenous Cx43 transcripts. In contrast, Cx43D cells displayed significantly higher Cx43 protein levels as demonstrated by western blotting (*p* < 0.05, Figure 1b) and by immunofluorescence (Figure 1c). Figure 1c shows a clear membranous co-localization of endogenous Cx43 with exogenous Cx43D, in Cx43D cells. Furthermore, the effect of Cx43 knock-down or over-expression on GJ functionality was assessed by fluorescence recovery after photobleaching (FRAP) assay. Fluorescence recovery in bleached cells was observed only in Cx43D cells, and not in control parental MDA-MB-231 and shCx43 cells (Figure 1d,e). These results validate that down- and up-regulation of Cx43 was achieved in shCx43 and Cx43D cells. In Cx43D, Dendra-2-Cx43 fusion protein co-localizes with endogenous Cx43 and forms functional GJs in these cells.

### 2.2. Cx43 Upregulation Decreases Formation of Invasive Cell Aggregates in 3D Cultures

**Figure 2:**
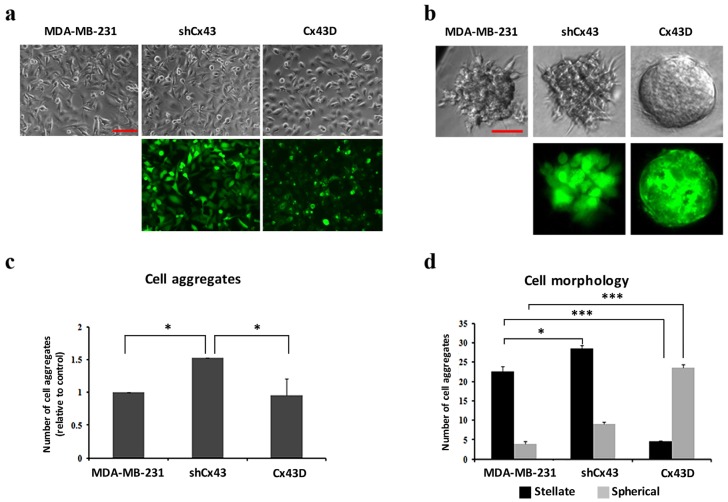
Cx43 upregulation decreases formation of invasive cell aggregates in 3D cultures. (**a**) and (**b**) Microscopic images for cells in 2D and 3D culture systems (scale bars of 100 and 50 µm), respectively. Upper panels show bright field images of cells/aggregates and lower panels show fluorescent images of shCx43 cells/aggregates or Cx43D cells/aggregates. (**c**) Bar graph showing the number of cell aggregates formed after 8 days in culture, normalized to number of cell aggregates formed by parental MDA-MB-231 cells. (**d**) Bar graph showing the numbers of cell aggregates with stellate *versus* spherical morphology after 8 days in culture. Results are averages of ten different fields of three independent experiments. * *p* < 0.05; *** *p* < 0.001.

In 2D culture, shCx43 cells maintained a mesenchymal-like phenotype, whereas Cx43D cells acquired a more epithelial phenotype (Figure 2a). In 3D culture, Cx43 knock-down induced a higher total number of cell aggregates (*p* < 0.05, Figure 2c). The proportion of stellate:spherical shCx43 cell aggregates was 3:1 (Figure 2b,d), characteristic of a greater invasive potential [39]. On the other hand, Cx43 over-expression favored cell aggregates with spherical morphology (Figure 2d), a result representative of what would be obtained using normal mammary epithelial cells, and a significantly lower proportion of stellate cell aggregates (*p* < 0.001, Figure 2d). Results of 3D cultures show a potential for Cx43 to suppress the malignant phenotype of breast cancer cells.

### 2.3. Cx43 Overexpression Decreases the Expression of EMT Markers

**Figure 3:**
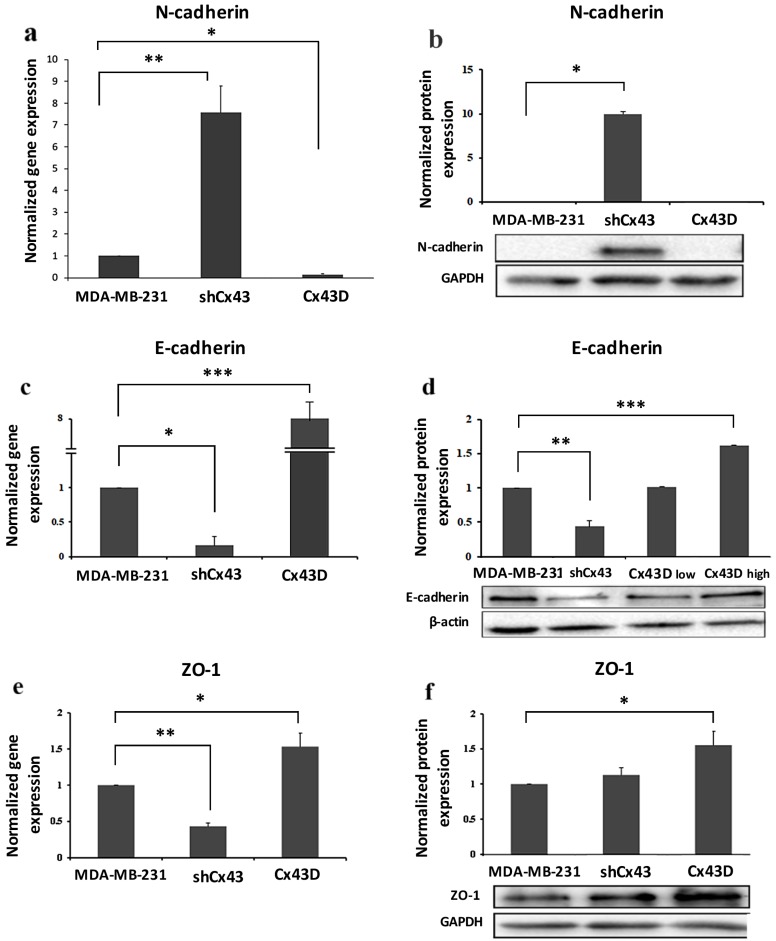
Cx43 overexpression decreases the expression of EMT markers. (**a**,**c**,**e**) Bar graph displaying N-cadherin, E-cadherin and ZO-1 mRNA expression levels in MDA-MB-231, shCx43 and Cx43D cells, as detected by qPCR and normalized to GAPDH. Results are representative of two independent experiments. (**b**,**d**,**f**) Western blots of N-cadherin, E-cadherin and ZO-1 protein expression in MDA-MB-231 cells, shCx43 and Cx43D cells, with densitometry analysis after normalization to GAPDH (or β-actin). Panel (**d**) also displays western blotting data of E-cadherin protein levels in Cx43D (low) and Cx43D (high). Results are representative of two independent experiments. * *p* < 0.05; ** *p* < 0.01; *** *p* < 0.001.

Phenotypic changes seen in shCx43 and Cx43D cells (Figure 2a) prompted the investigation of EMT markers; the expression levels of mesenchymal (N-cadherin) and epithelial markers (E-cadherin and zonula occludens 1 [ZO-1]) were assessed. N-cadherin mRNA levels were significantly higher in shCx43 cells (*p* < 0.001) than in both control parental MDA-MB-231 cells or Cx43D cells (Figure 3a). N-cadherin protein expression was significantly induced in shCx43 cells (*p* < 0.05), but not in control parental MDA-MB-231 cells or Cx43D cells, where it was not detected (Figure 3b). 

Cx43 knock-down resulted in significant decrease in expression of epithelial markers, E-cadherin (*p* < 0.05) and ZO-1 (*p* < 0.01). Consistently, upregulation of Cx43 induced a significant increase of both E-cadherin (*p* < 0.05) and ZO-1 (*p* < 0.001) at the transcriptional level (Figure 3c,e). These findings also translated at the protein level (Figure 3d,f). Interestingly, E-cadherin protein levels, in two sorted populations of Cx43D that show high (Cx43D high) and low (Cx43D low) fluorescence intensity, proportionally increased with increased Cx43 levels (Figure 3d).

### 2.4. Cx43 Knock-Down Enhances the Invasion of MDA-MB-231 Cells

**Figure 4:**
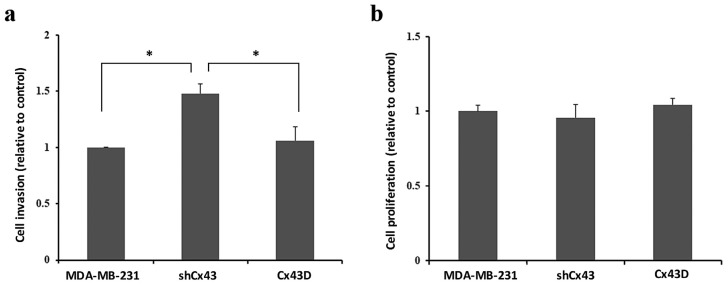
Cx43 knock-down enhances the invasion of MDA-MB-231 cells. (**a**,**b**). Bar graph showing the normalized cell index of shCx43 and Cx43D cells, relative to parental MDA-MB-231 cells, as detected by RTCA invasion and proliferation assays, respectively. Cell impedance readings were taken every 15 min for a minimum of 48 h. Results are representative of four independent experiments. * *p* < 0.05.

Enhanced expression of mesenchymal EMT markers in shCx43 could correlate with increased cell invasiveness [40]. Indeed, the invasive potential of MDA-MB-231 cells was increased by 30% upon knocking-down Cx43 (*p* < 0.05), as compared to control parental MDA-MB-231 cells or Cx43D cells (Figure 4a). Figure 4b shows that increased invasion was not due to increased proliferation.

### 2.5. Cx43 Over-Expression Sequesters β-Catenin at the Cell Membrane in MDA-MB-231 Cells

**Figure 5:**
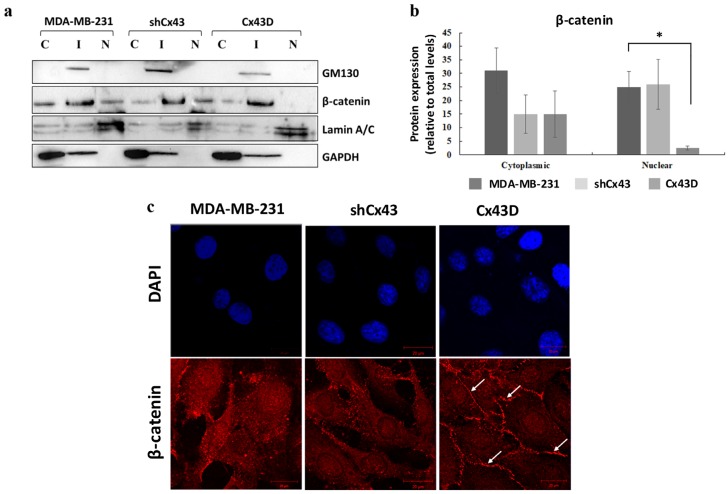
Cx43 over-expression sequesters β-catenin at the cell membrane in MDA-MB-231 cells. (**a**) Western blotting of β-catenin in cellular fractions of MDA-MB-231 cells, shCx43 cells and Cx43D cells. Proteins from the cytosolic (C), intermediate (I) and nuclear (N) fractions were blotted with compartment-specific antibodies, GAPDH, GM130 and Lamin A/C, respectively. (**b**) Bar graph displaying the densitometry analysis of nuclear versus cytosolic β-catenin expression with respect to total β-catenin protein levels. Two independent experiments were performed and plotted. (**c**) Representative immunofluorescence images of β-catenin expression in parental MDA-MB-231, shCx43 and Cx43D cells, counterstained with DAPI. Micrographs are representative of at least ten different fields per cell line. Scale bar represents 20 µm. * *p* < 0.05.

β-catenin is a member of the adherens junction complex and a transcription factor that regulates the expression of invasion, migration and proliferation genes. The expression and localization of β-catenin were compared in parental MDA-MB-231 cells, shCx43 cells and Cx43D cells. Levels of nuclear β-catenin protein were significantly decreased in Cx43D cells (*p* < 0.05, Figure 5a,b), compared to control parental MDA-MB-231 cells and shCx43 cells. Immunofluorescence showed that most of the β-catenin in Cx43D cells was mainly sequestered at the cell membrane (Figure 5c).

### 2.6. Cx43 Over-Expression Delays Tumor Onset, Decreases Tumor Volume and Increases Overall Survival

**Figure 6:**
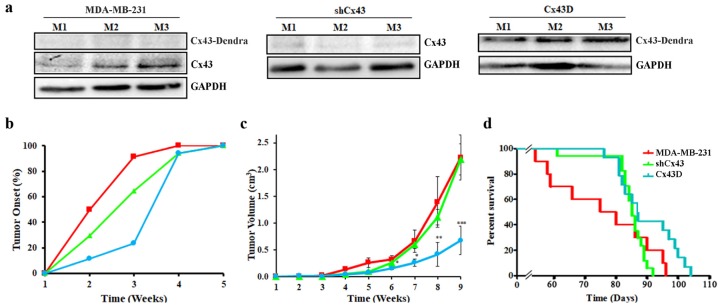
Cx43 over-expression delays tumor onset, decreases tumor volume and increases overall survival. (**a**) Western blotting of Cx43 in primary tumor tissues from three different mice, 9 weeks post-injection with parental MDA-MB-231, shCx43 or Cx43D cells. (**b**) Graph showing primary tumor onset over time. Mice were monitored for any palpable tumors at the site of injection. Percentage of mice with first palpable tumor occurrence was plotted against time. (**c**) Graph displaying tumor volume (in cm^3^) measured using a Vernier caliper, on a weekly basis throughout the experimental duration. (**d**) Kaplan-Meier survival curves of mice injected with parental MDA-MB-231, shCx43 or Cx43D cells. Mice were monitored daily until moribund state or death was reached. * *p* < 0.05; ** *p* < 0.01; *** *p* < 0.001.

In an effort to highlight the in vivo effects of Cx43 on cancer onset and progression, a xenograft mouse model of MDA-MB-231 cells with Cx43 knock-down or over-expression was used. As expected, primary tumors of mice xenografted with shCx43 cells showed down-regulated Cx43 protein levels as opposed to primary tumors of Cx43D cells that had upregulated Cx43 protein levels (Figure 6a). This result validates that the subsequent in vivo data are a consequence of variation in Cx43 expression levels.

Analysis of primary tumor occurrence and size showed that mice xenografted with Cx43D cells had a delayed tumor onset. More than 90% of the mice xenografted with parental MDA-MB-231 cells developed tumors by week 3, compared to 65% of mice xenografted with shCx43 cells and only 20% of mice xenografted with Cx43D cells (Figure 6b). Mice xenografted with Cx43D cells had a significant reduction of primary tumor volume starting week 6 (*p* < 0.05), becoming more pronounced at week 8 (*p* < 0.01) and week 9 (*p* < 0.001), whereas shCx43 cells xenografted mice showed similar primary tumor volume profile to mice injected with parental MDA-MB-231 cells throughout the experimental duration (Figure 6c). Furthermore, mice injected with Cx43D cells had a better survival rate compared to mice injected with shCx43 cells or parental MDA-MB-231 cells (Figure 6d). These data show that later tumor onset in mice bearing Cx43D cells correlates with smaller tumor volume and subsequently prolonged survival.

### 2.7. Down-Regulation of Cx43 Enhances Breast Cancer Metastasis to the Lung and Liver

**Figure 7:**
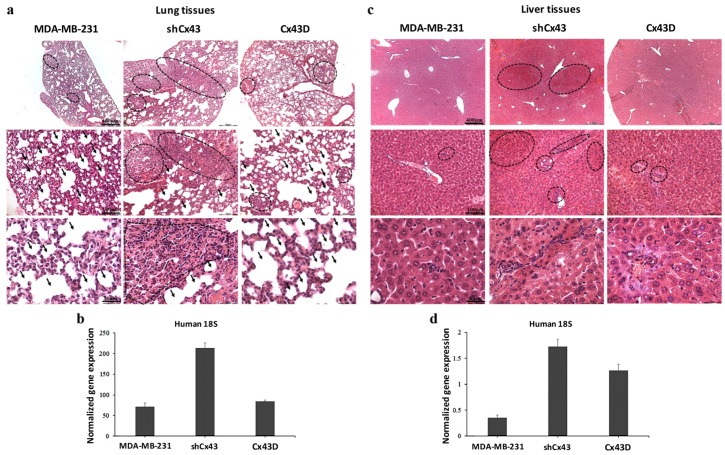
Down-regulation of Cx43 enhances breast cancer metastasis to the lung and liver. (**a**,**c**) H&E staining showing metastatic foci in lung and liver tissues, respectively, at week 9 post-injection of parental MDA-MB-231, shCx43 or Cx43D cells. (**b**,**d**) qPCR analysis of human 18S ribosomal RNA levels, in lung and liver tissues of mice bearing MDA-MB-231, shCx43 or Cx43D cells.

The above data suggest a protective role of Cx43 during breast cancer progression, evidenced as decreased tumor volume and prolonged survival. This protective effect was reflected by reduced metastasis to the lung and liver. Indeed, more metastases to the lungs were observed in mice injected with shCx43 tumor cells compared to parental MDA-MB-231 cells; shCx43 cells had almost filled the alveolar space by week 9 (Figure 7a). This observation was supported by qPCR analysis of human 18S RNA levels, at the secondary metastatic sites in the lungs. Higher levels of human 18S RNA were found in the lungs of mice injected with shCx43 cells compared to lungs of mice injected with parental MDA-MB-231 cells (Figure 7b). This observation indicates that down-regulation of Cx43 enhanced the metastatic potential of MDA-MB-231 cells (Figure 7b). Furthermore, by week 9, liver metastatic foci were almost absent in mice injected with parental MDA-MB-231 cells or Cx43D cells, while they were clearly observed in mice injected with shCx43 (Figure 7c). This result is in accordance with increased human 18S RNA levels in livers from mice inoculated with shCx43 cells (Figure 7d). In summary, down-regulation of Cx43 induced metastasis of MDA-MB-231 cells to the lung and liver at week 9, when parental MDA-MB-231 cells had not metastasized yet. These data correlated with increased tumor volume and a decrease in the survival rate of xenografted mice in vivo (Figure 6).

### 2.8. Cx43 Expression is Down-Regulated in TNBC Patients

**Figure 8:**
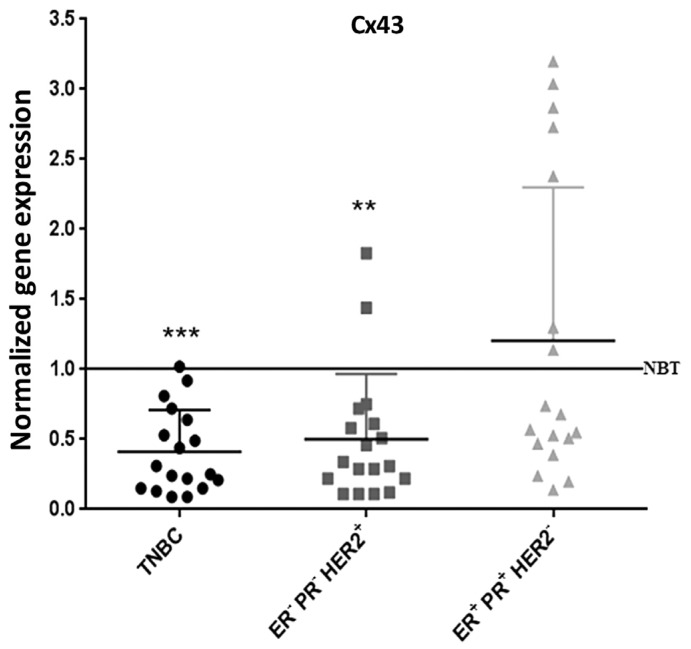
Cx43 expression is down-regulated in TNBC patients. qPCR analysis of Cx43 in TNBC, ER^−^ PR^−^ HER2^+^ and ER^+^ PR^+^ HER2^−^ subtypes of human breast cancer, after normalization to GAPDH. Results are plotted as fold change normalized to the expression level of Cx43 mRNA in normal breast tissues (NBT). ** *p* < 0.01; *** *p* < 0.001.

These data were further supported by assessing Cx43 levels in human breast cancer tissues. Transcriptional levels of Cx43 were determined in 54 randomly selected human breast carcinoma samples, distributed into three groups (18 samples per group); group 1: ER^−^ PR^−^ HER2^–^ (TNBC samples), group 2: ER^−^ PR^−^ HER2^+^, and group 3: ER^+^ PR^+^ HER2^−^. Cx43 mRNA levels were significantly lower in 95% of the TNBC samples (*p* < 0.001) and 90% of the ER^−^ PR^−^ HER2^+^ samples (*p* < 0.01), as compared to Cx43 mRNA levels of samples obtained from normal breast tissues (Figure 8). However, only 60% of the ER^+^ PR^+^ HER2^−^ samples showed a down-regulation in Cx43 expression, and the remaining 40% of samples in this group showed Cx43 up-regulation (Figure 8). Therefore, Cx43 was significantly down-regulated in virtually all of the highly aggressive breast cancer subtypes (TNBC and ER^−^ PR^−^ HER2^+^), comprising two-thirds of the total tested samples, and 60% of the less aggressive subtype. This finding corroborates in vitro and in vivo data reported above.

## 3. Discussion

This study investigated the effect of varying protein Cx43 levels on the metastatic potential of MDA-MB-231 TNBC cells, in vitro and in vivo, by either knocking-down (shCx43 cells) or over-expressing Cx43 (Cx43D cells) in these cells. In addition, the transcriptional expression levels of Cx43 in different types of human breast carcinoma tissues were investigated.

The regulation of genes/proteins implicated in progression, invasion and metastasis was assessed in vitro. Cx43 upregulation resulted in increased protein levels of epithelial markers E-cadherin and ZO-1 and the sequestration of β-catenin at the cell membrane, while Cx43 silencing induced protein expression of the mesenchymal marker N-cadherin and enhanced the invasiveness of MDA-MB-231 cells. In vivo results showed the attenuation of primary tumor growth and metastatic potential of breast cancer cells after Cx43 over-expression, whereas Cx43 knock-down induced a more aggressive metastatic phenotype.

MDA-MB-231 cells acquired a more mesenchymal phenotype upon knocking-down Cx43 and a more epithelial phenotype upon Cx43 over-expression. This called for the investigation of EMT marker expression. Indeed, Cx43 down-regulation resulted in expression of mesenchymal marker N-cadherin, while Cx43 over-expression promoted expression of epithelial markers E-cadherin and ZO-1. These findings are in line with a study reporting the domination of an epithelial phenotype upon Cx43 over-expression [23]. Therefore, the cadherin switch from E- to N-cadherin upon knocking-down Cx43, confirms the morphological observations and establishes the role of Cx43 in EMT of MDA-MB-231 cells. Accordingly, a recent study reports Cx43 as a direct transcriptional regulator of N-cadherin [41]. Increased EMT and increased expression of mesenchymal markers such as N-cadherin are correlated with increased cell invasiveness [40]. In fact, knocking-down Cx43 resulted in increased invasion of MDA-MB-231 cells (Figure 4), in combination with increased formation of stellate cell aggregates (Figure 2), indicative of enhanced metastatic potential [39]. On the other hand, Cx43 upregulation favored the formation of spherical cell aggregates, closer to an epithelial-like phenotype [39]. Malignant and invasive breast tumors are associated with mutations and over-expression of β-catenin. Upon Cx43 over-expression, β-catenin is reported to leave the nucleus and mainly translocate to the cell membrane [22]. These data are consistent with a model where high levels of membranous Cx43 recruit β-catenin to cell adhesion junction complexes [42,43], thus impeding its role as a transcriptional activator of genes involved in tumor progression and metastasis. β-catenin localization at the cell membrane favors mesenchymal-to-epithelial transition (MET), since several studies report that β-catenin nuclear localization is required for EMT [44,45] and that the β-catenin/TCF/LEF protein complex directly regulates genes associated with EMT, such as the EMT transcription factor Snail1 [46]. So Cx43 can regulate EMT genes indirectly by interacting with other proteins such as β-catenin or directly as reported by Kotini et al. [41]. Additionally, the increased levels of E-cadherin in MDA-MB-231 cells with upregulated Cx43 can explain the membranous localization of β-catenin in these cells. β-catenin localizes to the cell membrane in normal epithelial cells and in non-invasive tumor cells, whereas it localizes either in the cytoplasm (when dissociated from E-cadherin or Cx43) or in the nucleus, where it acts as a transcriptional activator of target genes including EMT genes, in cells that undergo EMT [47]. Taken together, these results would indicate greater metastatic potential of MDA-MB-231 cells with down-regulated Cx43 [39].

The enhanced invasive phenotype of MDA-MB-231 cells in vitro was consistent in vivo. Mice injected with MDA-MB-231 cells with up-regulated Cx43 had delayed primary tumor onset, smaller primary tumor volume and longer survival rate. These data match with the less invasive, more epithelial phenotype accompanied by upregulated MET genes observed in vitro [40]. We report a tumor-suppressive role of Cx43 during cancer progression that is in accordance with the literature [10,11,13,23,24].

Conversely, metastases of MDA-MB-231 cells to lungs and livers were promoted when Cx43 was down-regulated. These results are in correlation with a study showing that upon mutations and down-regulation of Cx43 in a breast cancer mouse model, earlier palpable tumors and a more aggressive cancer cell infiltration to the lungs were observed [26]. Cx43 down-regulation in MDA-MB-231 cells also supports a tumor suppressor activity of Cx43 at primary tumor sites [25,26]. Brain metastases, on the other hand, were reportedly promoted by Cx43-based gap junctions forming between MDA-MB-231 BrM2 cancer cells, a brain metastatic variant of MDA-MB-231 cells generated by Massagué and his group [48], and astrocytes in a mouse model where MDA-MB-231 BrM2 cells were injected in the left cardiac ventricle of mice [49]; thus proposing a brain-protective role for pharmacological inhibition of gap junction formation in the brain of patients with advanced breast cancer. Although breast cancer cells metastasize to the brain of patients with advanced breast cancer [50], organs most prone to breast cancer metastasis are bone, liver, lungs and then brain. Our study examined metastasis of MDA-MB-231 cells to the lungs and liver only.

Collectively, these data also support the notion of a spatio-temporal regulation of connexins expression during the different stages of metastasis and highlight their complex role in the tumorigenic process, where inversely a cancer-promoting role for connexins has been described [19,29,30,31,32,33,34,35,36,49].

To further explore the significance of our in vitro and in vivo findings in a clinical context, we evaluated the transcriptional expression of Cx43 in human breast carcinoma tissues of different subtypes, according to the expression of ER, PR and HER2, markers that predict the prognosis of breast cancer and guide the patients selection for therapy. In this study, 95% of human TNBC and 90% of ER^−^ PR^−^ HER2^+^ samples showed decreased Cx43 expression. In total, two-thirds of the 54 tested breast cancer samples showed a significant reduction in Cx43 transcription. Above all, virtually 100% of the more aggressive tumors showed reduced Cx43 mRNA levels, versus 60% of the less aggressive subtype. This finding was also demonstrated in another study that showed a direct correlation between Cx43 and ER/PR status in human breast carcinoma samples, however, it did not address Cx43 expression in TNBC nor in HER2^+^ samples, the most aggressive subtypes of breast cancers [51]. Laird et al. reported an absence of Cx43 expression in human breast carcinoma samples ranging from ductal carcinoma in situ, invasive lobular carcinoma and invasive ductal carcinoma. There was no differential Cx43 expression between the different subtypes and a correlation between Cx43 and ER/PR/erbB2 could not be established in the examined tissues [28]. Consistently, Busby et al., 2018 report that Cx43 expression depends greatly on intrinsic subtype [52]. Most importantly, Arora et al., 2018 report that decreased GJA1 expression leads to worse survival outcomes in patients, in several cancers including breast cancer [53], and another recent study reported Cx43 as an independent predictor of breast cancer patient prognosis and outcome [54].

## 4. Materials and Methods

### 4.1. Cell Culture

MDA-MB-231 cells were grown in RPMI-1640 media (Sigma, St. Louis, MO, USA) supplemented with 10% heat-inactivated fetal bovine serum (FBS; Sigma) and 1% penicillin-streptomycin (Sigma), and incubated at 37 °C in a humidified incubator (95% air, 5% CO_2_). Of the available human cell lines, MDA-MB-231 cells are the most commonly used model of TNBC, as they are widely considered to best represent TNBC. In fact, MDA-MB-231 cells are poorly differentiated, they exhibit wild-type BRCA1 and PI3K pathway genes and mutated KRas [39]. They are a highly metastatic triple negative human mammary adenocarcinoma cell line, invasive in vitro and readily incorporated into xenograft models.

MDA-MB-231 cells with down-regulated Cx43 (referred to as shCx43 cells thereafter) were also used. For the production of shCx43 cells, *pGFP-V-RS* vector with an inserted shRNA to silence Cx43 (OriGene Technologies, cat#: TR30007, Rockville, MD, USA) was transfected into MDA-MB-231 cells using Lipofectamine 2000 reagent (Invitrogen, Carlsbad, CA, USA) according to manufacturer’s instructions. OriGene Technologies supplied four different vectors with four different shRNA constructs that silence Cx43. All constructs have been validated by OriGene Technologies to have over 70% gene silencing. We have tested all four shRNA vectors and used the most efficient shRNA construct against Cx43. The *pGFP-V-RS* contains a GFP expression cassette, so transfected GFP^+^ cells were enriched by FACS sorting in a FACS Aria III SORP (BD Biosciences, San Jose, CA, USA). Following sorting, cells stably expressing shCx43 were selected using 0.5 µg/mL puromycin and then maintained in 0.1 µg/mL puromycin. The GFP-negative cell population was also recovered and used as negative control cell (sham cells).

MDA-MB-231 cells overexpressing Cx43 in fusion with Dendra-2, a photo-convertible fluorescent protein from green to red (referred to as Cx43D cells thereafter), were also used. Cx43D cells express a fusion protein of Dendra-2 fused at the N-terminus of Cx43; the Dendra-2-Cx43 fusion protein forms functional GJs [55]. To generate Cx43D cells, a *pCSCW-Dendra2-Cx43* lentiviral vector was co-transfected with packaging plasmids, using the calcium phosphate method, into HEK293T cells, for the production of viral particles that were collected in the supernatant 48 and 72 h post-transfection. MDA-MB-231 cells were transduced with lentiviral particles and sorted using FACS Aria III SORP (BD Biosciences) into ‘low’ or ‘high’ Dendra-2 expressing cells according to their fluorescence intensity. Cx43D high cells were used throughout the experiments, as Cx43D unless otherwise indicated.

### 4.2. Migration, Invasion and Proliferation Assays

Real-Time Cell Analysis (RTCA) was used to study invasion and proliferation of MDA-MB-231, shCx43, and Cx43D cells. Cells were seeded on a cell invasion plate (CIM-plate 16) with micro-electronic sensors on the underside of an 8-µm microporous polyethylene terephthalate membrane of a Boyden-like upper chamber, and real-time evaluation of cell impedance was performed using xCELLigence RTCA [A2] DP instrument (Roche, Mannheim, Germany). For invasion assays, 30 µL of growth factor-reduced Matrigel^TM^ (BD Biosciences) diluted in serum-free medium at a ratio of 1:20 was used to coat the upper surface of the membrane, followed by incubation for 4 h in a humidified incubator, and then washed with PBS. FBS was added to the lower chamber as a chemoattractant. The plate was incubated for 1 h at 37 °C before cells were seeded in the upper chamber at a density of 2 × 10^4^ cells per 100 µL of serum-free media.

For the cell proliferation assay, cells were seeded in an E-plate as described above, at a density of 1 × 10^4^ cells with an additional 150 µL of media containing 5% FBS. Both invasion and proliferation were monitored by recording cell impedance every 15 min for a minimum of 48 h.

### 4.3. Three-Dimensional Cell Culture and Sphere Counting

For 3D cultures, cells suspended in Matrigel^TM^:serum-free media (1:1) were uniformly plated around the bottom rim of 24-well plates and then supplemented with 2% serum-containing media. Media were replenished every 2–3 days and cell aggregates were counted after 8 days and classified into spherical versus stellate morphology.

### 4.4. Fluorescence Recovery After Photobleaching

Confluent MDA-MB-231 cells seeded onto glass-bottom culture dishes (confocal dishes) were incubated for 1 h with 1 μM calcein-AM (Invitrogen, Carlsbad, CA, USA), a dye converted by intracellular esterases to a green fluorescent molecule unable to cross the plasma membrane except through gap junctions. Pre-bleach images were taken, and then a specific cell was photobleached at 10% laser power (488 nm) for 5 iterations at 10-s intervals, using a laser scanning confocal microscope (LSM 710, Carl Zeiss, Oberkochen, Germany). Fluorescence intensity was measured before, during, and after bleaching. Dye transfer from surrounding cells was recorded every 5 s for a total of 5 min. Fluorescence intensity of the regions of interest (ROI) was quantified over time and normalized to that of control, unbleached, calcein-loaded cells, as previously described [56].

### 4.5. RNA Extraction and Quantitative PCR

Total RNA from cells and tissues was extracted using NucleoSpin RNA II Kit (Macherey-Nagel, Düren, Germany) and TriZol (ThermoFisher, Waltham, MA, USA), respectively. For RNA extraction from formalin-fixed paraffin-embedded (FFPE) tissues, the RecoverAll Total Nucleic Acid Isolation Kit (Ambion, Foster City, CA, USA) was used. One µg of total RNA was reverse-transcribed into single stranded cDNA using RevertAid first strand cDNA synthesis kit (ThermoFisher, Vilnius, Lithuania). Quantitative PCR (qPCR) was performed using iQ SYBR Green Supermix in a CFX96 system (Bio-Rad Laboratories, Hercules, CA, USA) with the primers listed in Table 1. The ΔΔCq method was applied to calculate the relative fold change in gene expression after normalization to GAPDH.

### 4.6. Protein Extraction and Western Blotting

Following cell lysis in a buffer containing 126 mM Tris/HCl, 20% glycerol *v*/*v*, 40 mg/mL of sodium dodecyl sulphate (SDS), proteins were resolved on SDS-PAGE gels, transferred on PVDF membranes (Bio-Rad) and incubated with specific primary and then secondary antibodies. Primary antibodies for Cx43 (detecting endogenous and exogenous Cx43 protein fused to Dendra-2), N-cadherin, ZO-1 (Invitrogen, USA), E-cadherin (Cell Signaling, USA), β-catenin, GM130 and Lamin A/C (Santa Cruz Biotechnology, Santa Cruz, CA, USA), β-Actin and GAPDH (Sigma) were used at 1 µg/mL. Proteins were detected by chemiluminescence and protein bands were analyzed by densitometry using Image J software (https://imagej.nih.gov/ij/).

### 4.7. Cellular Fractionation

Cells were lysed in buffer containing 10 mM HEPES, 10 mM KCl, 1.5 mM MgCl_2_, 1 mM dithiothreitol at pH 7.9 and then centrifuged. The supernatant was collected as the soluble cytoplasmic fraction (C), and the pellet containing nuclei was lysed in buffer containing 20 mM Tris, pH 7.4, 100 mM Nalco, 1% Triton X-100, 0.5% sodium deoxy cholate, 0.05% sodium dodecyl sulfate at pH 7.4 and then centrifuged. The supernatant (intermediate fraction, I) was collected and the pellet was lysed in buffer containing 50 mM Tris, 150 mM Nalco, 0.5% NP-40, 0.5% Triton X-100 at pH 7.5 and then centrifuged to pellet the nuclear fraction (N). All centrifugations were performed at 400× *g* for 10 min at 4 °C, and all collected fractions were finally cleared by centrifugation at 10,000× *g* for 10 min at 4 °C.

### 4.8. Immunofluorescence

Cells on coverslips were fixed and permeabilized in ice-cold 70% ethanol and then blocked in 3% non-immune goat serum in PBS for 1 h in a humidified chamber. Cells were then incubated with β-catenin antibody (Santa Cruz Biotechnology, USA) or Cx43 antibody (Sigma, USA), followed by incubation with IgG-conjugated Texas Red secondary antibody (Invitrogen, USA). Nuclei were counterstained with Hoechst 33324 (Thermo Fisher, Molecular Probes, Eugene, OR, USA). Coverslips were mounted onto glass slides using Prolong Antifade (Molecular Probes) and slides were observed under the LSM 710 microscope.

### 4.9. Xenograft Mouse Model of Breast Cancer Metastasis

This study was approved by the Institutional Animal Care and Utilization Committee of the American University of Beirut (IACUCC#10-07-154). Immune-deficient NSG mice (NOD.Cg-*Prkdc*^scid^ *Il2rg*^tm1Wj1^/SzJ, Jackson Laboratory, Bar Harbor, ME, USA) were injected with 2 × 10^6^ MDA-MB-231 cells into the subcutaneous area of the neck region. Cells were washed twice with PBS, re-suspended in serum-free media and passed through a 40-μm cell strainer to remove any cell clumps before injection. This model induces growth of primary tumor at the injection site, which would subsequently metastasize to secondary sites, as described previously [18]. Experimental groups (20 mice per group) included mice injected with parental MDA-MB-231 cells (control), shCx43 cells or Cx43D cells. Subsets of mice from each group were used for primary tumor analysis (onset and tumor volume), survival studies and metastasis studies at week 9 post-grafting using molecular analysis and histological examination.

### 4.10. Histological Examination of Lung, Liver and Primary Tumor Tissues

The lung, liver and primary tumor tissues obtained from mice at week 9 post-grafting were divided into 2 parts: one part was fixed in formalin, embedded in paraffin, processed and stained with Hematoxylin and Eosin (H&E) for examination under light microscopy; and the other was snap frozen in liquid nitrogen and stored at −80 °C for RNA/protein extraction and analysis.

### 4.11. Survival Analysis and Tumor Volume Measurement

Tumor growth and progression were monitored by palpation and measurement of tumor size, on a weekly basis, by double-blinded operators. Tumor size was determined by Vernier caliper measurements and tumor volume (in cm^3^) was determined by the equation: V = π/6 abc where a = length; b = width; c = height [57]. Animals were monitored on a daily basis for survival endpoints. Survival was defined as time to moribund state (or death), at which point mice were euthanized. Survival curves were plotted using the Kaplan-Meier method.

### 4.12. Human Breast Cancer Samples

Breast carcinoma samples were collected from the Pathology Departments of American University of Beirut-Medical Center and Hammoud Hospital University Medical Center. This study was approved by the Institutional Review Board Committee (Ref#: PALM.FB.01). Patients were females with no prior therapy, selected according to the immunohistochemical tumor expression profile of ER, PR and HER2. TNBC tumors were negative for ER, PR and HER2 expression (group 1). Whereas the ER^−^ PR^−^ HER2^+^ group (group 2) represents the luminal aggressive category, ER^+^ PR^+^ HER2^−^ group (group 3) represents the luminal less aggressive phenotype. A representative FFPE tissue block from each case was obtained for molecular analyses. Negative controls were obtained from breast tissue of patients who underwent reduction mammoplasty.

### 4.13. Statistical Analysis

Results are reported as the mean ± standard error of the mean (SEM). Statistical significance was determined using a Student’s *t*-test. Differences between groups were assessed by two-way analysis of variance (ANOVA), followed by Tukey’s post hoc test. The *p* value was determined and *p* < 0.05, *p* < 0.01, or *p* < 0.001 (*, **, ***; respectively) were considered significant. Microsoft Excel (and GraphPad Prism software were used for statistical analysis.

## 5. Conclusions

This multi-pronged study demonstrates that over-expression of Cx43 decreases the metastatic potential of a mammary adenocarcinoma cell line (MDA-MB-231), thus providing evidence for a pivotal role of Cx43 in breast cancer metastasis, and supporting the potential targeting of connexins in breast cancer therapy.

## Figures and Tables

**Table 1:** List of primers.

Gene	Primer Sequence	Annealing temperature (°C)
hCx43	F: CTTCACTACTTTTAAGCAAAAGAG R: TCCCTCCAGCAGTTGAG	52
hE-Cadherin	F: CAGAAAGTTTTCCACCAAAG R: AAATGTGAGCAATTCTGCTT	58
hZO-1	F: CAGCCGGTCACGATCTCCT R: GTGATGGACGACACCAGCG	58
hGAPDH	F: TGGTGCTCAGTGTAGCCCAG R: GGACCTGACCTGCCGTCTAG	58
h18S	F: CAGCCACCCGAGATTGAGCA R: TAGTAGCGACGGGCGGTGTG	58
mGAPDH	F: CATGGCCTTCCGTGTTCCTA R: CCTGCTTCACCACCTTCTTGAT	58

Cx43: Connexin43; F: Forward primer sequence; GAPDH: Glyceraldehyde 3-phosphate dehydrogenase; h: human; m: mouse; R: Reverse primer sequence; ZO-1: Zonula occludens 1.
